# Editorial: Immune Aging: Implications for Transplantation

**DOI:** 10.3389/fimmu.2022.953185

**Published:** 2022-06-21

**Authors:** John R. Greenland, Stefan G. Tullius, Joanna Schaenman

**Affiliations:** ^1^Department of Medicine, University of California, San Francisco, San Francisco, CA, United States; ^2^Division of Transplant Surgery, Department of Surgery, San Francisco Veterans Affairs (VA) Health Care System, San Francisco, CA, United States; ^3^Division of Transplant Surgery & Transplant Surgery Research Laboratory, Brigham and Women’s Hospital, Harvard Medical School, Boston, MA, United States; ^4^Division of Infectious Diseases, Department of Medicine, David Geffen School of Medicine at University of California, Los Angeles (UCLA), Los Angeles, CA, United States

**Keywords:** immune aging, transplantation, CMV, epigenetics, chimerism

Aging is fundamental to the human condition, but the biologic and physiologic consequences of aging can be accelerated or decelerated. In this Research Topic, we observe insights into biological aging elucidated by a mismatch between recipient and allograft age.

## Allograft Age

The biological age of an allograft reflects the donor’s date of birth, subject to acceleration or deceleration from environmental and molecular drivers. This age is retained at the time of transplant and likely impacts graft survival through multiple mechanisms: Telomeres are nucleoprotein caps at the end of chromosomes that shorten with age through cell division and stress, and critically short telomeres lead to senescence and cell cycle arrest. Mackintosh et al. observed that telomere length in lung allografts sampled by brushings of the small and large airways was correlated with donor age (Mackintosh et al.). Here, donor smoking history was linked to an acceleration in allograft biological age, with one pack per day of smoking exposure nearly doubling the rate of telomere erosion. Similarly, age impacts DNA methylation patterns, which are reset during embryogenesis and undergo characteristic changes over time. Dugger et al. observed that lung epithelial cells, also derived by small airway brushing, have a pattern of epigenetic age determined by the donor birthdate. These lung allografts all came from non-smoking donors, but epigenetic age was greater in allografts that had experienced primary graft dysfunction, again suggesting that stressors can accelerate biological aging. Both of these studies were underpowered to show an association with chronic lung allograft dysfunction or survival, but nominal hazard ratios suggested that donor biologic age was associated with worse outcomes, as has been seen in other studies (1–3). As Lin et al. summarize, allograft age is associated with stem cell dysfunction, senescence, mitochondrial dysfunction, and chromosomal alterations that can drive functional decline in stem cell and solid organ allografts.

## Recipient Aging

Older transplant recipients experience increased incidence of infection, malignancy, and death compared with younger patients, likely as a result of immune senescence associated with aging (4). Epigenetics and telomere dysfunction also contribute to the biological age of the recipient’s immune system (5, 6), and there are additional immune-specific aging mechanisms. CMV is a particularly strong driver of accelerate immune age. Higdon et al. demonstrated an increase in phenotypically aged CD8+ T cells, as defined by telomere length, CD57 expression, and terminal differentiation, in CMV seropositive patients. Innate T cells can also demonstrate immune senescence. Daniel et al. used KIR, NKG2A, and Eomesodermin to define a subtype of senescent innate T cells, which also had features resembling senescent adaptive T cells (CD27-/CD28-) and were more common in CMV seropositive kidney transplant recipients, independent of chronologic age. Chronic kidney disease can also accelerate immune aging, as evidenced by increased terminal differentiation in both responder and regulatory T cells in pre- and post-kidney transplant recipients (Leonhard et al.).

Immune aging may affect responses to pathogens or the allograft. Donor-specific hypo-responsiveness has been seen in older patients and may explain decreased risk for rejection. Van der List et al. observed an association between T cell immunoglobulin and ITIM domain (TIGIT), a marker of exhaustion, and polyfunctional donor-reactive CD4+ T cells in a cohort of younger and older kidney transplant recipients (van der List et al.).

The ability to target and reverse immune senescence holds promise for the older patient with cancer or requiring hematopoietic cell transplantation (HCT). Lin et al. reviewed the impact of immune senescence on HCT recipients and opportunities for reversing senescence including dasatinib plus quercertin, anakinra, or mTOR inhibitors.

## Interactions Between Donor and Recipient Age

While allograft age is retained after transplantation, allograft and recipient age do not remain entirely separate. Tissue-specific stem cells can migrate from recipient to allograft and vice versa (7) leading to age chimerism. As Iske et al. describe, donor-derived senescent cells can also induce aging phenotypes in the recipient through the section of cytokines, chemokines, and matrix remodeling enzymes or through direct cell contact (). Even a small number of transplanted senescent cells can induce senescence in multiple organs throughout the recipient in experimental models. Youthful allografts could theoretically drive rejuvenation, but the data here are less clear.

## Clinical Implications and Future Research

Observations in transplantation demonstrate help us understand the biological basis of aging phenotypes and the extent to which they can be manipulated, with important implications for aging biology more generally. In the care of a transplant candidate or recipient, understanding the mechanisms behind immune senescence may facilitate the optimal selection and matching of organs and candidates to optimize outcomes, and suggest interventions to ameliorate vulnerability to infection without increasing rejection risk. Mounting evidence that biologic age can differ from chronologic age indicates that measuring biologic age in the allograft and through immunologic assessment in transplant candidates and recipients may provide actionable clinical data.

## Author Contributions

All authors conceptualized, wrote, edited, and approved this manuscript for publication.

## Funding

JRG is supported by the Veteran Affairs Office of Research and Development, CSR&D section (CX002011), the National Heart Lung and Blood Institute (HL151552), and the Cystic Fibrosis Foundation. SGT has been supported by grants from the NIH (R56AGO39449, R01AG064165 and U01AI132898). JS is supported by the National Institute of Aging (R21 AG055879-01A1) and the Mendez Transplant Institute.

## Conflict of Interest

The authors declare that the research was conducted in the absence of any commercial or financial relationships that could be construed as a potential conflict of interest.

## Publisher’s Note

All claims expressed in this article are solely those of the authors and do not necessarily represent those of their affiliated organizations, or those of the publisher, the editors and the reviewers. Any product that may be evaluated in this article, or claim that may be made by its manufacturer, is not guaranteed or endorsed by the publisher.
